# The Influence of Natural Barriers in Shaping the Genetic Structure of Maharashtra Populations

**DOI:** 10.1371/journal.pone.0015283

**Published:** 2010-12-20

**Authors:** Kumarasamy Thangaraj, B. Prathap Naidu, Federica Crivellaro, Rakesh Tamang, Shashank Upadhyay, Varun Kumar Sharma, Alla G. Reddy, S. R. Walimbe, Gyaneshwer Chaubey, Toomas Kivisild, Lalji Singh

**Affiliations:** 1 Centre for Cellular and Molecular Biology, Hyderabad, India; 2 Leverhulme Centre of Human Evolutionary Studies, University of Cambridge, Cambridge, United Kingdom; 3 Department of Archaeology, Deccan College Post-Graduate and Research Institute, Pune, India; 4 Department of Evolutionary Biology, Institute of Molecular and Cell Biology, University of Tartu and Estonian Biocentre, Tartu, Estonia; University of Poitiers, France

## Abstract

**Background:**

The geographical position of Maharashtra state makes it rather essential to study the dispersal of modern humans in South Asia. Several hypotheses have been proposed to explain the cultural, linguistic and geographical affinity of the populations living in Maharashtra state with other South Asian populations. The genetic origin of populations living in this state is poorly understood and hitherto been described at low molecular resolution level.

**Methodology/Principal Findings:**

To address this issue, we have analyzed the mitochondrial DNA (mtDNA) of 185 individuals and NRY (non-recombining region of Y chromosome) of 98 individuals belonging to two major tribal populations of Maharashtra, and compared their molecular variations with that of 54 South Asian contemporary populations of adjacent states. Inter and intra population comparisons reveal that the maternal gene pool of Maharashtra state populations is composed of mainly South Asian haplogroups with traces of east and west Eurasian haplogroups, while the paternal haplogroups comprise the South Asian as well as signature of near eastern specific haplogroup J2a.

**Conclusions/Significance:**

Our analysis suggests that Indian populations, including Maharashtra state, are largely derived from Paleolithic ancient settlers; however, a more recent (∼10 Ky older) detectable paternal gene flow from west Asia is well reflected in the present study. These findings reveal movement of populations to Maharashtra through the western coast rather than mainland where Western Ghats-Vindhya Mountains and Narmada-Tapti rivers might have acted as a natural barrier. Comparing the Maharastrian populations with other South Asian populations reveals that they have a closer affinity with the South Indian than with the Central Indian populations.

## Introduction

The Indian subcontinent is legendary for the cultural, linguistic and genetic diversity of its inhabitants. The contemporary populations of India offer a platform to study the wide range of disciplines *viz.* demography, history, linguistics and genetics. Genetic diversity in India can be understood as a result of long term large effective population size, a number of dispersal events and its unique social structure. Maharashtra is the western most state of India inhabited by several caste and tribal populations. It is politically bordered by Gujarat, Madhya Pradesh, Chhattisgarh, Karnataka and Andhra Pradesh in the northwest, northeast, east, south and southeast, respectively. The Arabian Sea makes up Maharashtra's west coast. There are three mountain ranges in this state viz. Sahyadri in west, Satpuda in north and Gondwan in the east. More importantly, the Western Ghats mountain ranges of India is considered as the most densely populated global biodiversity hotspot which has a mosaic of natural, semi-natural and agroecosystems in close proximity to one another [1].

In India, the Maharashtra state ranks second in population and third in area. It constitutes 9.33 percent of India's population (Census 2001). The tribal populations accounts to about 9.3 percent of the total population of the state (Census 2001). There are 47 scheduled tribal population groups in the state and the majority of them are inhabitants of these geographically difficult topography [2]. The major tribal populations of Sahyadri range are the Mahadeo Koli, Thakur, Katkari, Warli, Malhar Koli and Kokana group. Among Satpuda ranges, Bhil, Pawara, Korku and Tadvi are the major groups. The Madia, Gond, Pardhan, Halbi Otkar and Andha are found in the Gondwan range. These tribal groups differ from each other in various aspects, for instance their different cultural practices, marriage pattern and socioeconomic categories. The origin and migration of these groups are uncertain. As majority of these tribal groups living in the remote forest areas remain isolated from each other thus, minimizes the chances of gene flow among them.

Mitochondrial DNA (mtDNA) evidence has been argued to support the model according to which populations of South Asians can trace their origin back to the Out-of-Africa (OoA) dispersal along the southern coastal route approximately 60 KYA [3]–[5] that is arguably in line with archaeological evidence [6]. Based on similarities reported in engraved pieces found at Blombos, South Africa, to those of Patne in India, and cresentic blade and microblade forms reported in India and Sri Lanka to Africa strongly indicated a direct connection between early human colonists in Asia and their ancestors in Africa [6]. However, the recent archaeological research has raised question about this dispersal and hypothesized alternative route [7], [8]. There is no archaeological evidence concerning the Paleolithic settlement of Western Ghats region by early modern human. It has been suggested that the early human populations in South Asia avoided the Western Ghats region because of high rainfall and thick vegetation [9]. Alternatively, it was proposed that such highly rich vegetation zone might have attracted early human because of ease of resources and the lack of corresponding evidence were explained due to various reasons (*e.g.*, inadequate surveys or thick vegetation not allowing proper surveys, lithics being transported after use instead of being discarded) [10].

Previous genetic studies on South Asian populations have identified their genepool as a composite of lineages that have evolved locally since the OoA dispersal and those that have been introduced by a number of more recent dispersal events [4], [11]–[18]. The analyses of mtDNA, Y chromosome, and autosomal genes have agreed on that the contribution of western Eurasian gene flow to India is more pronounced in the modern populations than that from East and Central Asia whereas, the relative proportions of imported *vs* locally differentiated genes appear to be dependent on the particular locus and populations being examined. The geographical location of Maharashtra state makes it quite interesting to study the dispersal of modern humans in South Asia. Besides harboring such an important geographical position in Indian subcontinent, the origin and migration of several populations living in various regions in this state of India and their affinity with other contemporary South Asian population has not been explored, so far, at the high resolution level. The available genetic source of this region is poorly known due to less sample sizes and low resolution [19], [20]. Moreover, this state works as a bridge among central, northern and southern parts of India and might give some clues for the peopling of Indian subcontinent, placing it on the way of coastal route migration [3], [5]. Therefore, to shed more light on the origin of the Maharashtra population, we first analyzed the control-regions and partial coding-region sequence variations of mtDNA and 20 Y-SNP and 17 Y-STR markers of Y chromosome in two tribal populations (Mahadeo-Koli and Thakur) inhabited in westernmost coastal region of India and compared the results with published sources from contemporary populations [4], [11]–[14], [19]–[30]. Our results not only help to further understand the phylogenetic position of Maharashtra state in South Asia but also provide deeper insights into the origin of Western Ghat populations.

## Materials and Methods

### Sampling

About 8–10 ml of blood was collected from 185 healthy unrelated individuals belonging to two tribal populations (Mahadeo Koli n = 95, Thakur n = 90) residing in Thane district of Maharashtra (Fig. S1). This project has been approved by the Institutional Ethical Committee (IEC) of Centre for Cellular and Molecular Biology (CCMB) and the informed written consent was obtained from all the participants. DNA was extracted from whole blood by using the standard protocol [31].

### mtDNA typing

Polymerase Chain Reactions were carried out with 10 ng DNA in a 10 ul reaction volume with 1U of Taq DNA polymerase enzyme. Cycling conditions used were 94°C for 5 min, 35 cycles at 95°C for 30 s, 58°C for 30 s, and 72°C for 2 min, then 72°C for 7 min. Sequencing of the PCR products were directly carried out by using Big Dye™ Terminator cycle sequencing Kit (Applied Biosystems, Foster City USA) in 3730 DNA Analyzer, following manufacture's protocol. To minimize errors, both strands were double-sequenced. The individual mtDNA sequences were compared against the rCRS [32] using AutoAssembler-Ver 2.1 (Applied Biosystems, Foster City USA). The sequencing data corresponding to nucleotide positions 15927–16510 and 16520–400 that includes HVS-I and HVS-II of the control region were sequenced in 94 Mahadeo Koli and 90 Thakur samples. Further, 19 segments of coding regions (nps.14890–15430,14120–14950,13370–14150,11970–12720,11330–12080,10000–10780,8630–9390,7950–8710,7170–8040,5880–6650,5280–6030,4520–5330,3810–4620,3190–3870,2510–3300,1870–2670,1240–1980,630–1370) were sequenced for 184 samples. All the newly generated sequences has been deposited in the GenBank (accession number HQ427694–HQ427878).

### Y-chromosomal typing

Twenty Y chromosome biallelic polymorphic markers M89, YAP, M216, M130, M9, M45, M74, M52, M304, M172, M410, M67, M11, M20, M27, M175, M95, M173, M17, and M124 were typed. The PCR cycles were set-up with an initial denaturation of 5 min at 95°C, followed by 30–35 cycles of 30 sec. at 94°C, 30 sec at the primer-specific annealing temperature (52–60°C), and 45 sec. at 72°C, and final extension of 7 min at 72°C. Length variation at 17 Y-STR loci: DYS456, DYS3891, DYS390, DYS389II, DYS458, DYS19, DYS385a/b, DYS393, DYS391, DYS439, DYS635, DYS392, DYS437, DYS438, DYS448 and Y GATA H4 were genotyped using AmpFℓSTR® Y-filer™ PCR amplification Kit (Applied Biosystems). Multiplex polymerase chain reaction was carried out with 0.5ng template with 1 U AmpliTaq Gold DNA polymerase enzyme (Applied Biosystems, Foster City, CA) with total reaction volume of 6.25 µl which includes 10mM Tris-HCl (PH 8.3), 50mM KCl, 1.5mM Mgcl_2_, 250 µm dNTPs, 3.0 µm of each primer. The conditions for the polymerase chain reaction are (1) 95°C for 10 min, (2) 28 cycles; 94°C for 1 min, 55°C for 1 min, 72°C for 1 min, (3) 60°C for 45 min, and (4) 25°C hold. ABI 3730 DNA Analyser (Applied Biosystems, Foster City, CA) and GeneMapper V4.0 software program (Applied Biosystems, Foster City, CA) was used for the analysis of raw data.

### Phylogenetic and Statistical analysis

Principal component analysis (PCA) was performed using POPSTR, kindly provided by H. Harpending. Median-joining and reduced median joining networks were reconstructed with NETWORK program (version 4.1) (www.fluxus-engineering.com). Reduced median and median-joining procedures were applied sequentially. Coalescence time has been calculated between nucleotide positions 16090–16365 (HVS-I) considering one transition equals to 20,180 years [33]. Standard deviation of the rho estimate (σ) was calculated as in Saillard et al. [34]. The diversity indices including AMOVA (analysis of molecular variance) based *Fst* analysis were calculated using ARLEQUIN 3.01 software [35].

The age of a Ychromosomal haplogroup have been obtained by the TD statistic, assuming mutation rate of 6.9×10^−4^ [36]. Haplogroups carrying less than 13 samples has not been included in age calculation. Out of 17 loci obtained, two DYS385 loci were excluded from the current analyses because they could not be distinguished using the typing method employed. Thus, all the analysis linked with Y-STR data were carried out with 15 loci. Haplogroup isofrequency maps were generated by using Surfer 8 of Golden Software (Golden Software Inc., Golden, Colorado), following the Kriging procedure. The present data from two tribes (Mahadeo Koli and Thakur) of central India were compared with previously published datasets [4], [11]–[14], [19]–[28], [30].

## Results and Discussion

**Figure 1:**
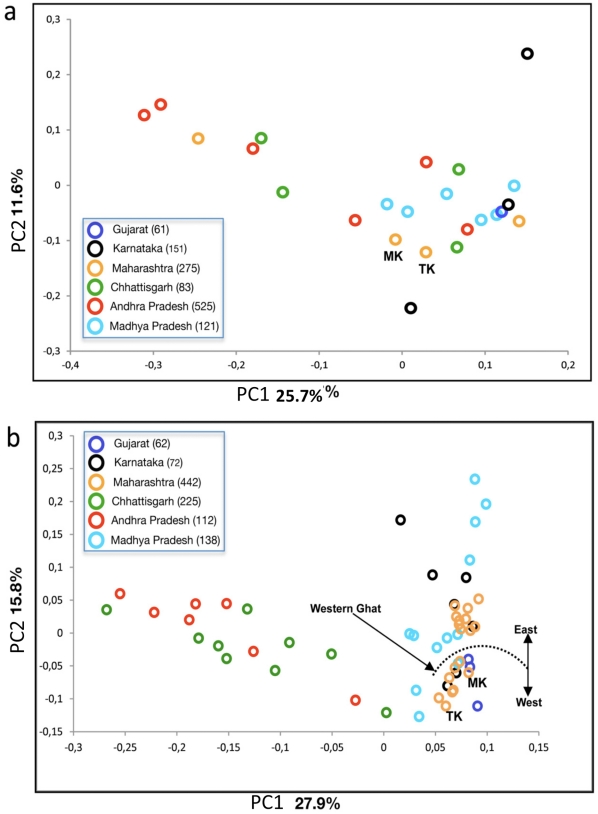
Principal component Analysis. Principal component (PC) scatter plot of mtDNA (a) and Y chromosomal (b) showing the genetic relation among population of Maharashtra and contemporary populations.

In order to understand the genetic relationship of populations of Maharashtra in the context of rest of the populations of India, we determined the summary genetic distances based on mtDNA and Y-chromosomal haplogroup frequencies, and subjected the resulting genetic distance matrices to principal component analysis and median joining network analysis (Fig. 1 and Fig. S2). The mtDNA PCA plot (Fig. 1a) did not show any clear-cut geographic or linguistic clustering in the data, while PC1 in the Y chromosomal plot (Fig. 1b) illustrates a distinct east to west clinal pattern. The Maharashtra populations cluster together and remain closer to South Indian and Gujarati populations than Central Indian populations (Fig. 1b). Within the Maharashtra populations we can see two clear-cut geographical clustering among the populations living on both sides of Western Ghat (Fig. 1b and Table 1). The most likely explanation of the above observations is the complex geographical structuring of this region. Populations living in Central India and in Maharashtra state are well separated by Narmada and Tapti rivers as well as Satpuda range of hills and populations living at the coastal region are equally separated from mainland populations by Sahyadri mountains (a range of Western Ghats), thus, restricting the easy population movement in either direction, while the long coastal region facilitates the gene flow from Karnataka and Gujarat states (Table 1). Therefore, geography is the main factor shaped the genetic composition of Maharashtra populations. Austroasiatic populations are the exceptions, who unanimously show their paternal affinity according to their linguistic division (Table 2). This is due to their highly frequent paternal haplogroup (hg) O2a [13], [15], [30].

**Table 1:** The Analysis of Molecular Variance (AMOVA) results.

	*Fst*	
	mtDNA	Y Chromosome
Maharashtra (east) Vs Madhya Pradesh	0.0537	0.2761
Maharashtra (east) Vs Andhra Pradesh	0.0411	0.1469
Maharashtra (east) Vs Karnataka	0.0492	0.0898
Maharashtra (east) Vs Gujarat	0.0501	0.1998
Maharashtra (west) Vs Madhya Pradesh	0.0794	0.4859
Maharashtra (west) Vs Andhra Pradesh	0.0567	0.3771
Maharashtra (west) Vs Karnataka	0.0293	0.0419
Maharashtra (west) Vs Gujarat	0.0173	0.0211

**Table 2:** The frequencies of different Y chromosomal haplogroups in Maharashtra populations, *n* is number of samples and * is the position relative to Western Ghat.

Population	Position*	Language	Social Status	*n*	F*(M89)	H(M69)	J(xJ2)	J2(M172)	L(M11)	O2a(M95)	O3(M122)	P*(M45)	R*(M207)	R1a1(M17)	R2(M124)	Reference
Maratha	East	Indo-European	Caste	53	0	0.39	0	0.15	0.06	0	0	0	0	0.19	0.06	Trivedi et al. 2008
Dhangar	East	Indo-European	Caste	33	0	0.38	0	0.06	0.06	0	0	0	0	0.31	0.18	Trivedi et al. 2008
Pawara	East	Indo-European	Tribe	16	0	0.75	0	0.06	0.06	0	0	0	0	0.06	0.06	Trivedi et al. 2008
Katkari	East	Indo-European	Tribe	19	0	0.63	0	0.05	0.05	0	0	0.05	0.05	0.16	0	Trivedi et al. 2008
Madia-Gond	East	Dravidian	Tribe	14	0	0.57	0	0	0.29	0	0	0.07	0	0	0.07	Trivedi et al. 2008
Maratha	East	Indo-European	Caste	20	0.05	0.35	0	0.15	0.15	0	0	0	0	0.10	0.20	Sengupta et al. 2006
Naba-Baudh	East	Indo-European	Religious group	14	0	0.43	0	0.14	0.07	0	0	0	0	0.21	0	Sengupta et al. 2006
Korku	East	Austroasiatic	Tribe	59	0.07	0.08	0	0	0	0.81	0.02	0.02	0	0.00	0	Kumar et al. 2007
Desasth Brahmin	West	Indo-European	Caste	35	0.05	0.13	0	0.18	0.11	0	0	0	0	0.37	0.16	Trivedi et al. 2008
Chitpavana Brahmin	West	Indo-European	Caste	39	0	0.23	0	0.17	0.13	0	0	0	0	0.27	0.20	Trivedi et al. 2008
Konkan Brahmins	West	Indo-European	Caste	23	0	0.08	0	0.16	0.04	0	0	0	0	0.48	0.20	Sengupta et al. 2006
Mahadeo-Koli	West	Indo-European	Tribe	50	0.24	0.18	0.04	0.14	0.02	0	0	0	0	0.26	0.12	Present Study
Thakur	West	Indo-European	Tribe	48	0.13	0.08	0	0.27	0.06	0	0	0	0	0.29	0.04	Present Study

**Figure 2:**
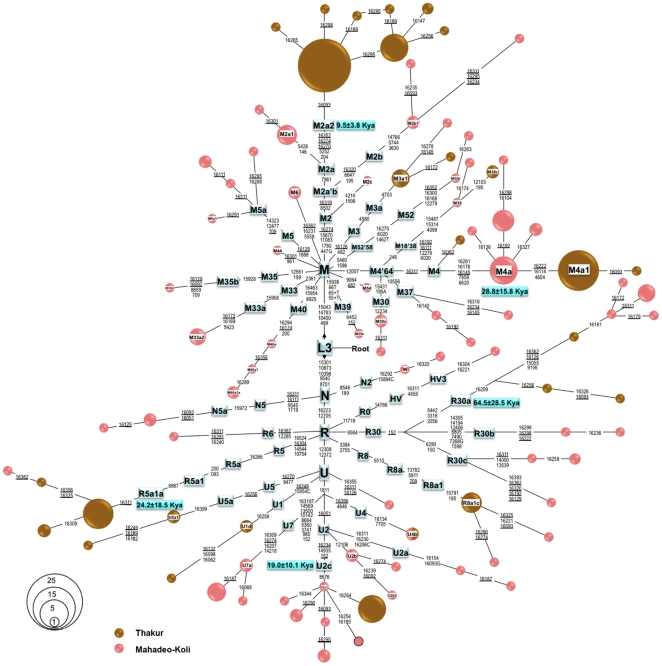
The reduced-median network of 185 mtDNA subjects belonging to Mahadeo-Koli and Thakur populations. This network was redrawn manually from the output of median joining/reduced network obtained using NETWORK program (version 4.1) (www.fluxus-engineering.com). Each sample represented on the diagram has been sequenced for the HVS-I region and genotyped for the coding region mutations that are indicated. Circle sizes are proportional to the number of mtDNAs with that haplotype. Recurrent mutations are underlined. Coalescent times were calculated by a calibration method described elsewhere [33]. 16182C, 16183C and 16519 polymorphisms were omitted. Suffixes A, C, G, and T indicate transversions.

The Network based analysis of mtDNA haplogroups in currently studied Mahadeo-Koli and Thakur populations identified several South Asian and a few West Eurasian specific sub-clades in the background of mtDNA macrohaplogroup M and N(R) (Fig. 2 and Table S1). The haplotype diversity in Thakur population was least among Maharashtra populations (Table 3). In macrohaplogroup M background haplogroups (hgps); M2, M3, M4 and M18 are shared by both Mahadeo-Koli and Thakur populations, while hgps M5, M6, M30, M33, M35, M37, M39, M40, M44 and M52 are exclusively present in Mahadeo-Koli. Two hgps of macrohaplogroup N (N5 and W) are present in Mahadeo-Koli while most of the macrohaplogroup R and U clades are shared by both of the populations, except hgps R6 and HV3 (Fig. 2).

**Table 3:** mtDNA diversity indices calculated in Maharashtra and neighbouring state populations.

Population	State	*n*	HD (SD)	MPD (SD)	ND (SD)
Andh	Andhra Pradesh	66	0.981 (0.005)	5.190 (2.544)	0.013 (0.007)
Brahmin	Andhra Pradesh	40	0.995 (0.007)	5.394 (2.655)	0.014 (0.007)
Chenchu	Andhra Pradesh	110	0.925 (0.011)	5.740 (2.770)	0.015 (0.008)
Erukala	Andhra Pradesh	27	0.966 (0.022)	5.325 (2.652)	0.014 (0.007)
Kapu	Andhra Pradesh	52	0.993 (0.005)	5.812 (2.824)	0.015 (0.008)
Koya	Andhra Pradesh	81	0.941 (0.016)	6.363 (3.047)	0.016 (0.008)
Lambadi	Andhra Pradesh	86	0.985 (0.005)	5.858 (2.826)	0.015 (0.008)
Naikpod	Andhra Pradesh	92	0.976 (0.005)	5.198 (2.538)	0.014 (0.007)
Thoti	Andhra Pradesh	39	0.906 (0.030)	5.519 (2.711)	0.015 (0.007)
Baiga	Chhattishgarh	26	0.910 (0.230)	5.592 (2.834)	0.014 (0.006)
Birhor	Chhattishgarh	20	0.891 (0.002)	5.363 (2.149)	0.015 (0.007)
Gujarati	Gujarat	57	0.996 (0.004)	6.223 (2.999)	0.017 (0.008)
Lobana	Gujarat	61	0.913 (0.033)	6.268 (3.127)	0.017 (0.011)
Koragas	Karnataka	51	0.988 (0.066)	5.895 (2.754)	0.015 (0.008)
BettuKurumba	Karnataka	79	0.899 (0.078)	6.912 (3.397)	0.014 (0.007)
Kanwar	Madhya Pradesh	19	0.965 (0.036)	5.556 (2.792)	0.014 (0.008)
Mushar	Madhya Pradesh	46	0.852 (0.041)	6.719 (3.227)	0.017 (0.009)
Satnami	Madhya Pradesh	18	0.974 (0.025)	5.301 (2.684)	0.014 (0.007)
Bhar	Madhya Pradesh	22	0.922 (0.045)	4.208 (2.171)	0.011 (0.006)
Harijan	Madhya Pradesh	20	0.990 (0.019)	5.326 (2.683)	0.014 (0.007)
Mahadeo-Koli	Maharashtra	95	0.990 (0.003)	6.454 (3.082)	0.017 (0.009)
Thakur	Maharashtra	90	0.903 (0.020)	6.353 (3.039)	0.016 (0.008)
Maratha	Maharashtra	30	0.995 (0.010)	6.749 (3.273)	0.017 (0.009)
Chitpawana Brahmin	Maharashtra	20	0.979 (0.026)	6.389 (3.159)	0.017 (0.009)
Desastha Brahmin	Maharashtra	19	1.000 (0.017)	5.895 (2.944)	0.015 (0.008)
Dhangar	Maharashtra	19	1.000 (0.017)	6.327 (3.139)	0.016 (0.009)
Konkan Brahmin	Maharashtra	58	0.985 (0.008)	6.059 (2.927)	0.016 (0.008)

HD = Haplotype Diversity.

MPD = Mean number of pairwise difference.

ND = Nucleotide Diversity.

The high resolution analysis at haplogroup and sub-haplogroup level identified a monophylacity of previously classified M2a and M2b subclades with a single coding as well as control region substitution and named as M2a'b (Fig. 2). This finding recognizes a sub-branch M2c and refines the defining mutations for haplogroup M2 [37], [39]. It is now defined by four coding and single control region mutations (Fig. 2). Similarly the finding of several branches in the background of haplogroups M4, R5, R8 and R30 has improved the resolution of mtDNA phylogeny of this region. It is noteworthy that although Thakur and Mahadeo-Koli live in a close proximity, there is no haplotype sharing among them, except single haplotype share in West Eurasian haplogroup U4 (Fig. 2). This suggests a high level of strict endogamy in these two populations regardless both being at the same social level and exchange the rituals and other traditional occupations with one another and maintain their unique identity. It is consistent with the previous observation on South Asian populations [15]. The coalescent age of different mtDNA lineages are calculated in the studied populations (Fig. 2). Majority of the sub-clades have a coalescent time ranging from 10–30 KYA (Fig. 2).

The Y chromosome analysis identified nine major haplogroups in Maharashtra populations (Table 2), of which South Asian specific haplogroup H is most frequent in caste and tribal populations. Second most frequent haplogroup is hg R1a present in caste as well as tribal populations. Some of the studies considered hg H as a tribal and hg R1a as caste specific marker previously [12], [24], [25]. In contrast to them, the present study supports the occurrence of these haplogroups in both caste and tribal populations of India [11], [15]. The discrepancy of frequency distribution of these haplogroups in caste and tribal populations can be explained by their different population sizes where evolutionary forces act in a different way and diverse social customs that involve practicing endogamy at different levels [18].

**Figure 3:**
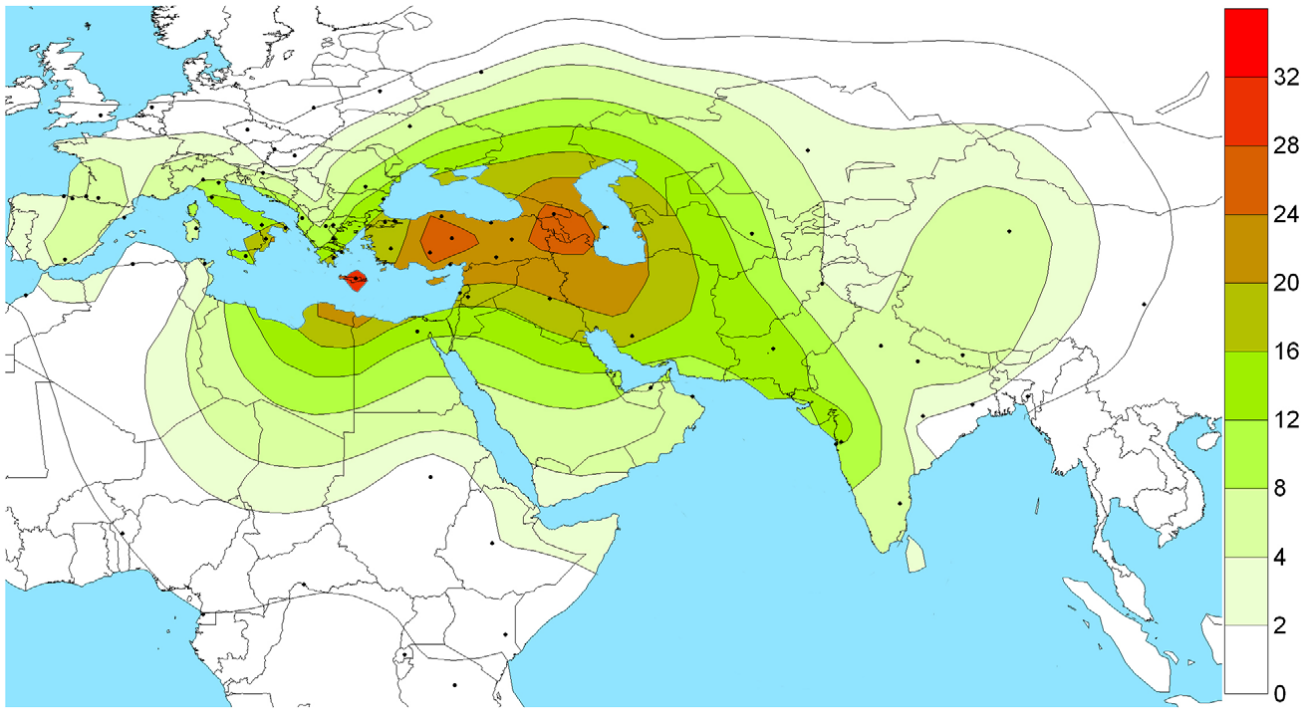
The spatial distribution (%) of M410(J2a) clade in worldwide populations. Isofrequency maps were generated by using Surfer8 of Golden Software (Golden Software Inc., Golden, Colorado), following the Kriging procedure. Data is used from present study and previously published literatures [14], [23], [26]. The dots represent sampling locations.

**Figure 4:**
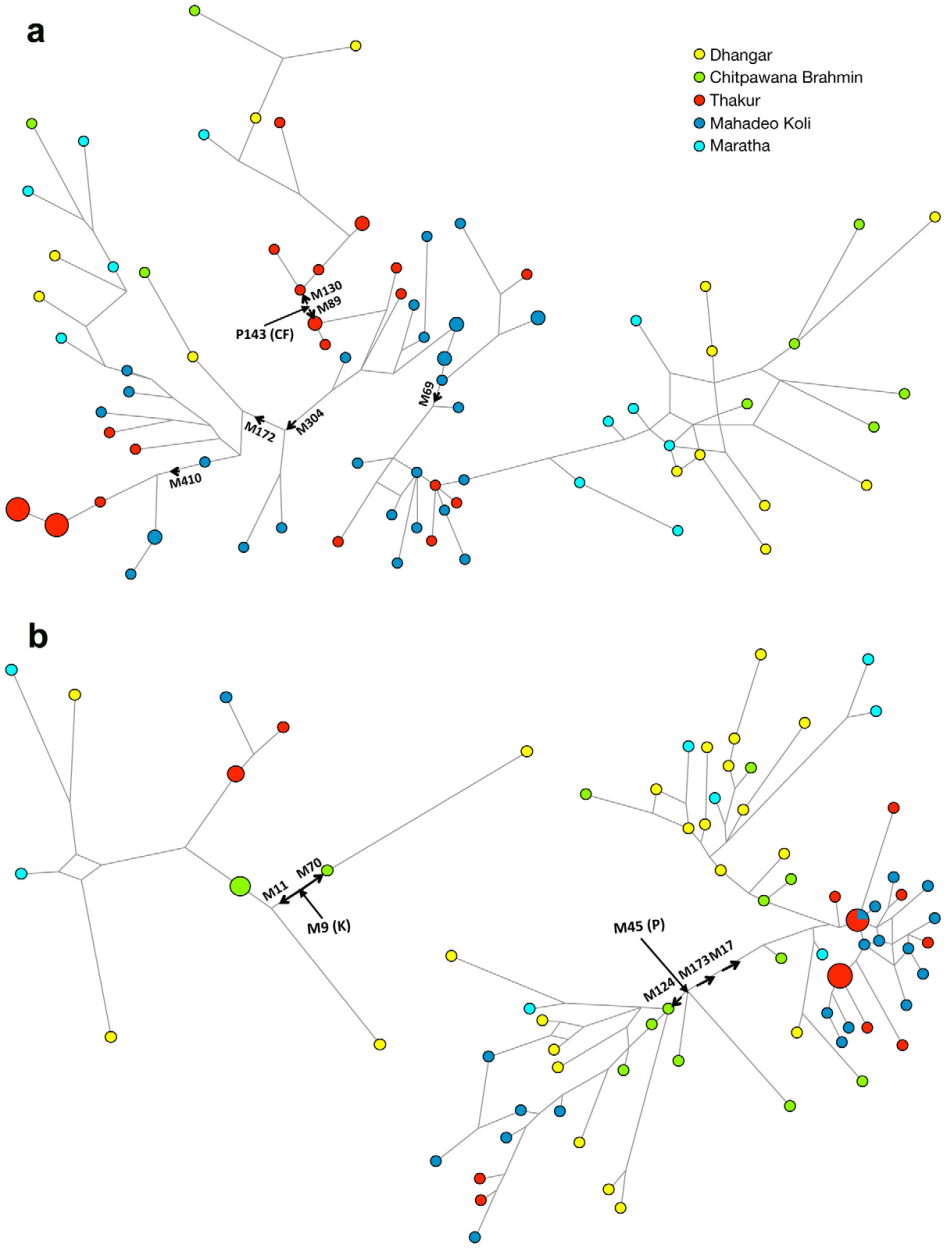
Rooted phylogenetic network of Maharashtra populations. (a) Rooted phylogenetic network of Maharashtra populations relating Y-STR haplotypes within macrohaplogroup CF (P143) and its associated phylogeny: The network was constructed using a median-joining algorithm as implemented in the Network 4.5.0.2 program. The size of the circles is proportional to the number of samples. (b) Rooted phylogenetic network of Maharashtra populations relating Y-STR haplotypes within macrohaplogroup K(M9) and M45(P) with their associated phylogeny. The network was constructed using a median-joining algorithm as implemented in the Network 4.5.0.2 program. The size of the circles is proportional to the number of samples.

Near Eastern specific hg J2 is also significantly present in both of the studied populations (Table 2 and Table S2). This haplogroup thought to be associated with the intrusion from Near East during Neolithic agricultural expansion [14]. Further dissection of this hg revealed most of the samples to be derived for marker M410 (hg. J2a). The further genotyping of M410 derived samples remained ancestral to M67 marker (hg. J2a4). The worldwide phylogeographic distribution of hg J2a suggests its entry in Indian subcontinent through northwestern corridor and an abrupt drop further south due to Western Ghat mountain ranges (Fig. 3). The rooted Y-STR network of different Y chromosomal haplogroups provided a diverse haplotype distribution in Maharashtra populations (Fig. 4).

By using the Y-STR data from both of the populations, we have calculated the variance and coalescent ages for different haplogroups (Table 4). The age of microsatellites variation in all of the major haplogroups ranges from 7–35 KYA (Table 4). The South Asian specific haplogroups F*, H1a and R2 show pre-Neolithic, while hg L1 shows Neolithic expansion time. The age of haplogroup R1a ranges from 10–17 KYA which is consistent with previous large scale study on this haplogroup [38]. The network analysis of R1a with other Indian populations failed to provide any regional or linguistic clustering (Fig. S2).

**Table 4:** The coalescent age and variance of different Y chromosomal haplogroups observed in studied populations.

Haplogroup	Sample size	Age (Kya)	Variance
F*	18	26,3±4,8	0.66
H1a	13	17,65±3,9	0.45
J2a	15	15,3±7,6	0.36
R1a	27	14,1±3,2	0.34

In conclusion, our results on Maharashtra populations are consistent with other Indian populations suggest that the tribal as well as caste populations of Indian subcontinent practice a strict endogamy even though they live in a close proximity and share the ritual and social customs. The mtDNA results dissected and increased the clarity of South Asian mtDNA phylogeny. The colonization of western part of Western Ghat is facilitated mainly through migration of populations via western coast rather than mainland where Western Ghat-Vindhya mountains and Narmada-Tapti rivers worked as a natural barrier. Our data is in congruent with the other observations that Indian populations including Maharashtra state are largely derived from Paleolithic ancient settlers, however, a more recent (∼10 Ky older) detectable paternal gene flow from west Asia is well reflected in present genetic study.

## Supporting Information

Figure S1 The sampling location of Mahadeo Koli and Thakar populations.
(TIF)

Figure S2 Unrooted phylogenetic network of haplogroup R1a Y-STR haplotypes among different Indian populations showing the haplotype sharing of Thakur and Mahadeo-Koli. The network was constructed using a median-joining algorithm as implemented in the Network 4.5.0.2 program. The size of the circles is proportional to the number of samples.
(TIF)

Table S1 The complete mtDNA data from Mahadeo-Koli and Thakur populations.
(XLS)

Table S2 The Y-SNP and Y-STR complete data from Mahadeo-Koli and Thakur populations.
(XLS)
