# Supplementary figures and images for: The Influence of Natural Barriers in Shaping the Genetic Structure of Maharashtra Populations
Source: PLoS One. 2010 Dec 20;5(12):e15283. doi: 10.1371/journal.pone.0015283 (PMC3004917; doi:10.1371/journal.pone.0015283)

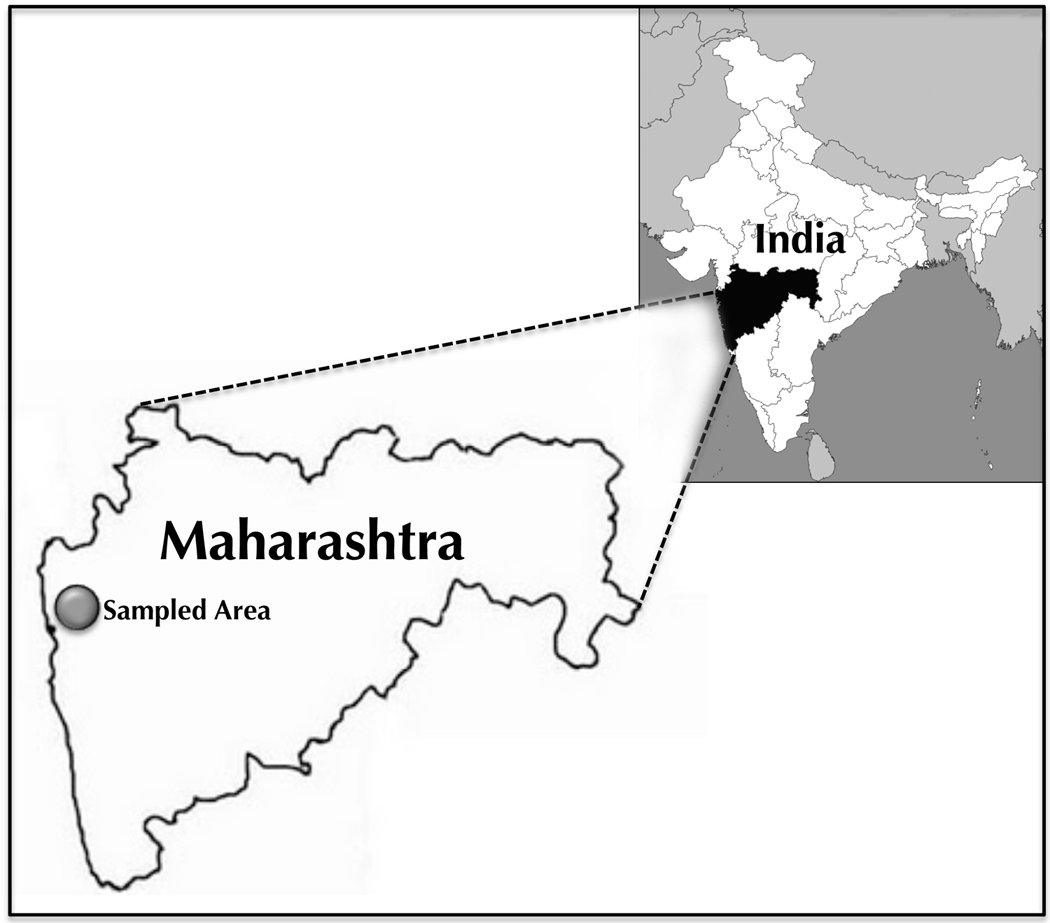

Supplement: Figure S1 — The sampling location of Mahadeo Koli and Thakar populations. (TIF) [file pone.0015283.s001.tif]

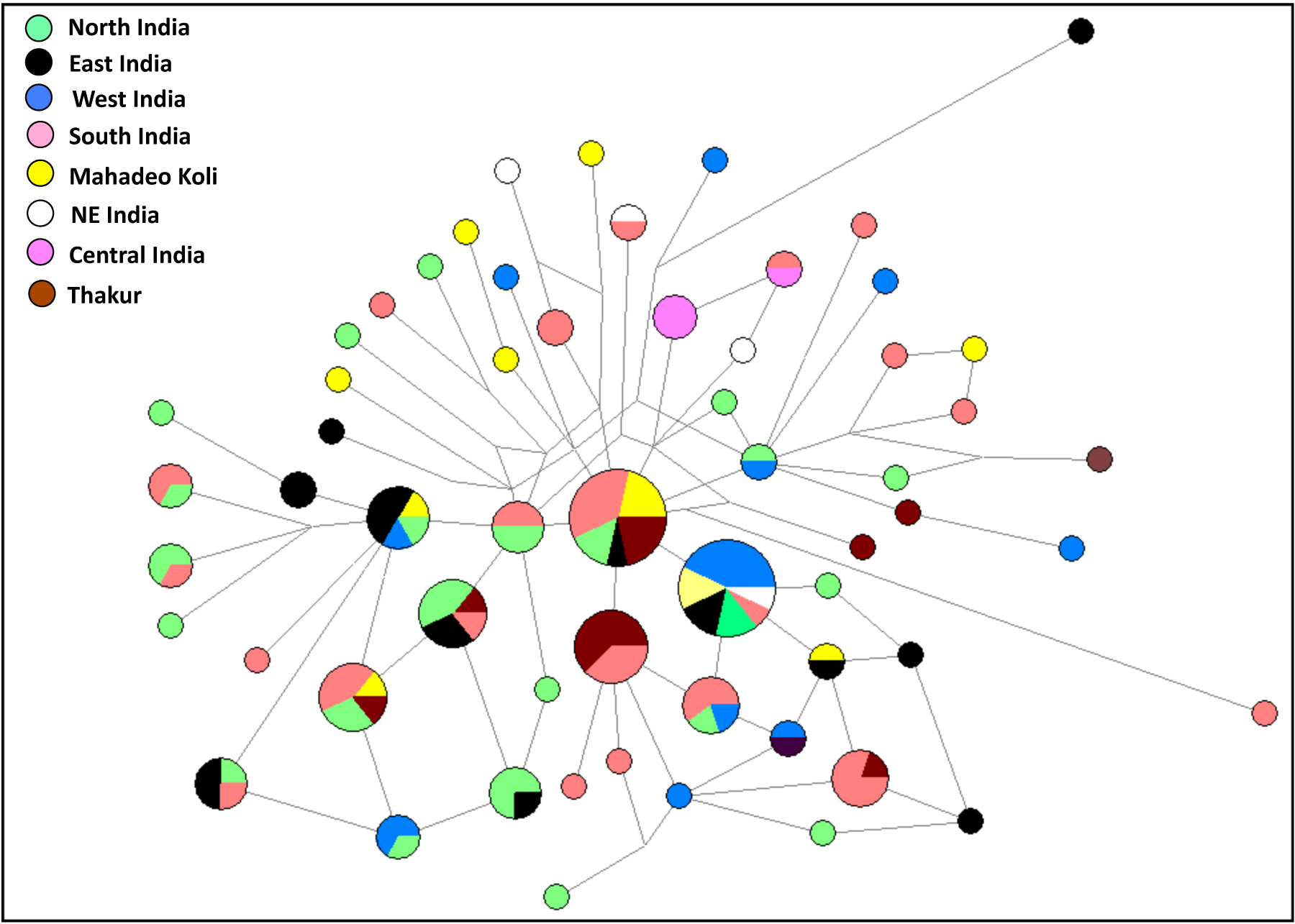

Supplement: Figure S2 — Unrooted phylogenetic network of haplogroup R1a Y-STR haplotypes among different Indian populations showing the haplotype sharing of Thakur and Mahadeo-Koli. The network was constructed using a median-joining algorithm as implemented in the Network 4.5.0.2 program. The size of the circles is proportional to the number of samples. (TIF) [file pone.0015283.s002.tif]
